# Atorvastatin and Fenofibrate Increase the Content of Unsaturated Acyl Chains in HDL and Modify In Vivo Kinetics of HDL-Cholesteryl Esters in New Zealand White Rabbits

**DOI:** 10.3390/ijms20102521

**Published:** 2019-05-22

**Authors:** Cristóbal Flores-Castillo, María Luna-Luna, Elizabeth Carreón-Torres, Victoria López-Olmos, Sara Frías, Marco Antonio Juárez-Oropeza, Martha Franco, José Manuel Fragoso, Gilberto Vargas-Alarcón, Óscar Pérez-Méndez

**Affiliations:** 1 Department of Molecular Biology, Instituto Nacional de Cardiología “Ignacio Chávez”, 14080 Mexico City, Mexico; flockmx@gmail.com (C.F.-C.); mjluna.qfb@gmail.com (M.L.-L.); elizact73@gmail.com (E.C.-T.); vickyolmosunam@hotmail.com (V.L.-O.); mfragoso1275@yahoo.com.mx (J.M.F.); gvargas63@yahoo.com (G.V.-A.); 2 Laboratorio de Citogenética, Instituto Nacional de Pediatría, 04530 Mexico City, Mexico; sarafrias@biomedicas.unam.mx; 3 Departamento de Medicina Genómica y Toxicología Ambiental, Instituto de Investigaciones Biomédicas, Universidad Nacional Autónoma de México, 04510 Mexico City, Mexico; 4 Department of Biochemistry, School of Medicine, Universidad Nacional Autónoma de México, 04510 Mexico City, Mexico; majo_ya@yahoo.com.mx; 5 Department of Nephrology, Instituto Nacional de Cardiología “Ignacio Chávez”, 14080 Mexico City, Mexico; marthafranco@lycos.com

**Keywords:** statins, fibrates, HDL metabolism, dyslipidemia, atherosclerosis

## Abstract

Previous studies demonstrated modifications of high-density lipoproteins (HDL) structure and apolipoprotein (apo) A-I catabolism induced by the atorvastatin and fenofibrate combination. However, it remains unknown whether such structural and metabolic changes of HDL were related to an improvement of the HDL-cholesteryl esters (HDL-CE) metabolism. Therefore, we determined the structure of HDL and performed kinetic studies of HDL-CE radiolabeled with tritium in rabbits treated with atorvastatin, fenofibrate, and a combination of both drugs. The atorvastatin and fenofibrate combination increased the HDL size and the cholesterol and phospholipid plasma concentrations of the largest HDL subclasses. Moreover, the relative amount of unsaturated fatty acids contained in HDL increased, in detriment of saturated fatty acids as determined by gas chromatography–mass spectrometry. The transfers of cholesteryl esters (CE) from HDL to very low-density lipoproteins/low-density lipoproteins (VLDL/LDL) and vice versa were enhanced with atorvastatin, alone or in combination. Moreover, the direct elimination of CE from plasma via VLDL/LDL decreased with fenofibrate, whereas the direct elimination of CE via HDL augmented with the combination treatment. Taken together, the rise of unsaturated fatty acid content and the size increase of HDL, suggest that atorvastatin and fenofibrate induce more fluid HDL particles, which in turn favor an enhanced CE exchange between HDL and VLDL/LDL. Our results contribute to a better understanding of the relationship between the structure and function of HDL during the use of anti-dyslipidemic drugs.

## 1. Introduction

The negative correlation between high-density lipoproteins-cholesterol (HDL-C) and the development of cardiovascular disease is well known [1,2]. Therefore, different pharmacological interventions have been focused on the elevation of the HDL-C, but such interventions have been ineffective in the reduction of cardiovascular risk [3,4,5,6]. This apparent paradox may be partially explained by the fact that HDL-C does not reflect the complex composition of the high-density lipoproteins (HDL) particles and their functionality [7,8].

HDL constitute a heterogeneous group of lipoproteins structured mainly by apolipoprotein (apo) A-I, cholesterol, cholesteryl esters (CE), triglycerides, and phospholipids. HDL may be classified by their size in five subclasses (HDL2b, HDL2a, HDL3a, HDL3b, and HDL3c), which differ in lipids, proteins [8,9], and probably anti-atherogenic functions [8,10]. HDL subclasses may be influenced by several factors including ethnicity [11], triglycerides plasma concentrations [12], antidiabetic or lipid-lowering drugs [13,14], and pathological conditions; particularly, low-large and high-small HDL subclasses have been observed in adults and children with insulin resistance [15,16]. Therefore, the structure of HDL has been related to their metabolism and functionality. Notably, the HDL structure may reflect the metabolic activity of adipose tissue [17,18].

The transport of cholesterol from the foam cells to the liver known as reverse cholesterol transport (RCT), has been considered the principal anti-atherogenic function of the HDL [19,20]. The RCT begins with the cholesterol efflux from peripheral cells to lipid-poor HDL; then the cholesterol is esterified and exchanged for triglycerides in apo B-containing lipoproteins, which are eliminated from plasma via the hepatic LDL-receptor. Alternatively, high-density lipoproteins-cholesteryl esters (HDL-CE) may be directly internalized into the cytoplasm of hepatocytes and then recycled, stocked up in the endoplasmic reticulum, transformed to bile salts, or secreted in bile without biochemical modifications. In this regard, the cholesterol efflux of patients with coronary heart disease has been inversely associated with cardiovascular events [21] but, HDL has other anti-atherogenic functions also related with the structure of these lipoproteins, including anti-inflammatory, anti-oxidant, anti-aggregant, and profibrinolytic properties [2,8,22].

The potential relationship between the structure and function of HDL is controversial and has not yet been fully understood. Accordingly, size changes of HDL particles have been positively associated with an improved cholesterol efflux capacity in older adults [23]. Moreover, the shift of fatty acids profile from saturated to unsaturated has been associated with more fluid HDL particles [24], with enhanced capacity as cholesterol acceptors [25]. Conversely, the impaired cholesterol transport characteristic of patients with metabolic syndrome has been associated with HDL particles enriched with triglycerides [26] and saturated fatty acids [27]. Furthermore, the transport of cholesterol seems to be independent of the HDL-C plasma concentrations [17] and the cholesterol efflux is decreased in patients with both, coronary heart disease and increased HDL-C levels [27]. Taken together, the evidence suggests that the lipid and protein composition of HDL is a better risk marker of cardiovascular disease than the HDL-C levels, and this raises the need to elucidate the structure and metabolism of functional HDL in terms of cardiovascular protection.

In this context, we previously demonstrated that the atorvastatin and fenofibrate combination modified the lipid composition and increased the size and apo A-I catabolism of the HDL particles in New Zealand white rabbits [28]. However, it is unknown whether such structural changes of HDL include modifications of the fatty acids content and whether they are related to an improvement of the HDL-CE transport in vivo. Therefore, we determined the lipid composition of HDL and performed in vivo kinetic studies of HDL-CE radiolabeled with tritium in rabbits treated with atorvastatin, fenofibrate, and the combination of both drugs. Our results demonstrated different changes of the HDL structure induced by atorvastatin, fenofibrate, and the combination treatment as well as the impact of such changes on the kinetics of HDL-CE.

## 2. Results

### 2.1. Biochemical Analyses

The total cholesterol and glucose plasma concentrations were similar among the atorvastatin, fenofibrate, combination, and control rabbits. However, the fenofibrate treatment tended to decrease the triglycerides (Table 1). The HDL-C and high-density lipoproteins-phospholipids (HDL-Ph) plasma concentrations of rabbits treated with the combination were 44% and 24% higher, respectively, than those of the control rabbits (Table 1). In addition, there were differences of HDL-C and HDL-Ph plasma concentrations between the rabbits treated with atorvastatin or fenofibrate in comparison with the group that received the combination treatment. Interestingly, apo A-I plasma concentrations increased 55% and 41% in the rabbits that received the fenofibrate and the combination treatment, respectively, with respect to the control rabbits (Table 1).

### 2.2. Structure and Lipid Composition of HDL

**Figure 1:**
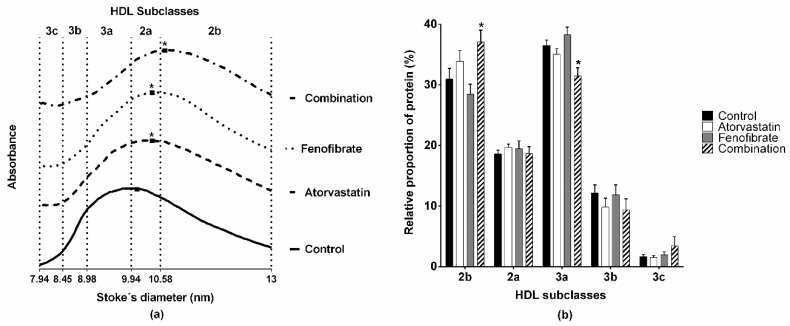
The atorvastatin and fenofibrate combination treatment increased the size of the high-density lipoprotein (HDL) particles. HDL were isolated by ultracentrifugation and separated in subclasses by non-denaturing 3%–30% polyacrylamide gel electrophoresis. The gels were stained for protein and were scanned to obtain the data to construct the graphs. (**a**) Graphs of HDL obtained from control, atorvastatin, fenofibrate, and combination rabbits. *n* = 6. Arbitrary absorbance units are plotted on the vertical axis. (**b**) The relative HDL size distribution estimated from graphs. The total area under the curve was considered as 100% and the partial areas between the intervals as relative percentages of HDL subclasses. Data are mean ± standard error. *n* = 6. * ANOVA test, *p* < 0.05 vs. control rabbits.

We determined the size and lipid composition of HDL of rabbits by non-denaturing 3%–30% polyacrylamide gel electrophoresis; the densitometric analysis of gels showed a displacement of the maximal point of absorbance towards large HDL subclasses of either the group that received atorvastatin or fenofibrate, as compared with the control group (Figure 1a, atorvastatin and fenofibrate at 10.39 ± 0.08 nm vs. control at 10.06 ± 0.04 nm, *p* = 0.011). The most important displacement to larger diameters was observed for the group that received the combination of atorvastatin and fenofibrate (Figure 1a, maximal point of absorbance 10.67 ± 0.10 nm, *p* < 0.001 vs. control). Consistently, the HDL size distribution of combination rabbits shifts towards the largest HDL subclass (HDL2b, Figure 1b). Furthermore, the HDL size distribution of the rabbits that received only atorvastatin or fenofibrate was similar to that of the control rabbits (Figure 1b).

Regarding the cholesterol plasma concentrations of the HDL subclasses, they were similar between the rabbits that received only atorvastatin or fenofibrate in comparison with the control rabbits (Table 2). However, the cholesterol plasma concentration was higher by 40% for the HDL2b, HDL2a, and HDL3a in the combination group with respect to that of the control rabbits (Table 2). Likewise, the combination treatment increased by 35% and 15% of the phospholipids plasma concentration of the HDL2b and HDL2a with respect to the control group. Additionally, there were differences in the phospholipids of the HDL2b and HDL2a subclasses of the rabbits treated with the combination as compared to the rabbits treated with the single drugs. Further, there were no changes in the triglycerides plasma concentrations of HDL subclasses among the four groups of rabbits (Table 2).

### 2.3. Fatty Acids Composition of HDL

**Figure 2:**
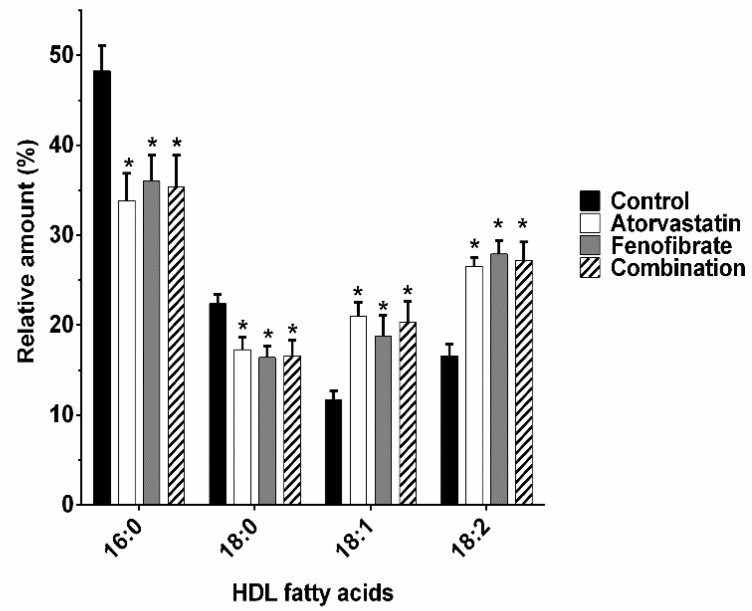
The relative amount of total HDL fatty acids shifted from saturated fatty acids towards unsaturated fatty acids with atorvastatin, fenofibrate, and the combination treatment. HDL were used to extract the lipids and the fatty acids composition was determined by gas chromatography–mass spectrometry. The fatty acids were identified by their retention time and mass spectrum; 16:0, palmitic acid; 18:0, stearic acid; 18:1, oleic acid; 18:2, linoleic acid. Data are mean ± standard error, *n* = 6. * ANOVA test, *p* < 0.05 vs. control rabbits.

Recent reports indicate that changes in the composition of HDL fatty acids may affect their cholesterol transport function. Therefore, we determined the composition of HDL fatty acids. The relative amount of saturated fatty acids decreased meanwhile the relative amount of unsaturated fatty acids increased in the HDL of three groups of treated rabbits; palmitic acid (16:0) decreased by 30%, 25%, and 27% in the HDL of rabbits treated with atorvastatin, fenofibrate, and the combination, respectively, compared to that of the control rabbits (Figure 2). Correspondingly, the relative amount of stearic acid (18:0) decreased by 24%, 27%, and 26% in the HDL of rabbits treated with atorvastatin, fenofibrate, and the combination, respectively, compared to that of the control rabbits (Figure 2). In contrast, the relative amount of oleic acid (18:1, Δ^9^) increased 79%, 61%, and 74% in the HDL of rabbits treated with atorvastatin, fenofibrate, and the combination, respectively, compared to that of the control rabbits and the relative amount of linoleic acid (18:2, Δ^9,12^) increased by 60%, 68%, and 64% in the HDL of all treated rabbits with respect to that of the control group (Figure 2).

### 2.4. In Vivo Kinetic Studies of HDL-CE

To explore whether the modifications of HDL structure have an effect on the cholesterol transport, we performed in vivo kinetic studies of HDL-CE. The exchange of CE between lipoproteins was highly increased in the treated rabbits. The net transfer of CE mass from the HDL to VLDL/LDL fraction was increased about seven- and four-fold in the rabbits with the atorvastatin and combination treatments, respectively, than that in the control rabbits. Similarly, the net transfer of CE from the VLDL/LDL to HDL fraction was approximately 7-fold higher in the rabbits treated with atorvastatin and the combination than that in the control rabbits (Table 3). The fenofibrate treatment increased only three and two times the bidirectional transfer of CE between lipoproteins (i.e., transfer from HDL to VLDL/LDL and from VLDL/LDL to HDL, respectively) but the difference with the control group values did not reach statistical significance (Table 3). Additionally, the elimination of CE via HDL (i.e., HDL outflow) was approximately two-fold higher in the rabbits treated with the combination of atorvastatin and fenofibrate in comparison with that in the control group (Table 3). In contrast, the elimination of CE via VLDL/LDL (i.e., VLDL/LDL outflow) decreased by half with the fenofibrate treatment with respect to that of atorvastatin and control groups (Table 3).

### 2.5. Activity of the Cholesteryl Ester Transfer Protein (CETP)

To explore the possibility that the increased transfer flows rates of CE between lipoproteins were due to the CETP, we determined the activity of this protein in the plasma of all rabbits. The activity of CETP was similar among all the treated and control rabbits (25.02 ± 3.03%, 24.48 ± 2.32%, 25.12 ± 2.82%, and 23.10 ± 3.78% for atorvastatin, fenofibrate, combination, and control rabbits, respectively, *p* > 0.05).

## 3. Discussion

The ineffectiveness of the pharmacologic treatments intended to elevate the HDL-C plasma concentrations [3,4,5,6] and reducing cardiovascular risk indicates that HDL-C does not reflect the lipoprotein functionality. Instead, the structure of HDL (i.e., size distribution, lipid and protein content), is a better marker of the anti-atherogenic properties of these lipoproteins [7]. A recent study strongly suggests that the increase of brown adipose tissue metabolism in mice induced by cold exposition or pharmacological thermogenic activation is linked to an enhanced cholesterol efflux through the HDL and to the disposal of cholesterol in feces [17]. Importantly, the increased brown adipose tissue in that study was associated with significant modifications of the proportional content of lipids within HDL, whereas HDL-C plasma concentrations remained unchanged. Hence, there is an important connection between a cardio-protective metabolism (i.e., increased brown adipose tissue) and the HDL structure and turnover. In agreement with this idea, the present study demonstrated changes in the HDL structure induced by two cardio-protective drugs, atorvastatin, fenofibrate, and the combination of both, and the effect of such changes on the kinetics of HDL-CE in vivo.

New Zealand white rabbits have been widely used to evaluate the kinetics of lipoproteins [13,28,29,30,31,32,33,34] due to their metabolic similarities with humans, which are absent in other animal species such as rats and mice [35]. Previously, we reported that the HDL structure is importantly modified in the setting of exogenous hypercholesterolemia [28]. However, hypercholesterolemia was not completely reverted by statins at used doses [28] and possibly, remaining dyslipidemia may be a confusing factor for the CE kinetic studies. Therefore, in this study we used only normal rabbits instead of rabbits with dyslipidemia to follow the transfer of CE between lipoproteins and their subsequent elimination from plasma. Moreover, we used ponderal doses of the drugs comparable to those prescribed in humans, which are at least ten times lower than that used in other animal studies [33,36,37,38].

Herein, we reported a huge increase of the bidirectional flow rates of CE between lipoproteins (i.e., HDL to VLDL/LDL and VLDL/LDL to HDL) in the treated rabbits. The net transfer of CE mass from the VLDL/LDL to HDL was highly increased in the two groups of rabbits with atorvastatin treatment (alone or in combination), whereas fenofibrate did not have a significant effect on such CE exchange. Hence, atorvastatin enhanced the transfer of CE mass from proatherogenic to antiatherogenic lipoproteins, which agrees with the cardioprotective action of statins [39].

Concerning the elimination of CE from plasma, the atorvastatin and fenofibrate combination increased the outflow of CE mass via HDL. It is known that fibrates, as well as statins, increase the expression of the scavenger receptor class B, type I (SR-BI) [35,40,41], which has been proposed to be selective for the uptake of HDL-CE by peripheral cells [42]. Therefore, the enhanced elimination of CE from plasma via HDL may be a consequence of a synergic effect of the combination of atorvastatin and fenofibrate on the SR-BI expression. Nevertheless, other potential mechanisms of elimination of HDL-CE may not be discarded; an increased transfer of HDL particles to the interstitial compartment [43], an increased activity of lipoprotein lipase [17], and/or an SR-BI-independent delivery of HDL-CE to peripheral cells [44] may occur.

Contrary to the HDL outflow, the elimination of HDL-CE from plasma via VLDL/LDL decreased with the fenofibrate; this result is conflicting with the expected effect of PPARα agonists on the expression of VLDL and LDL receptors [45,46]. It should be considered that our study was performed in vivo, and the observed results are the balance between the production, exchange among lipoproteins, and the elimination from the plasma of CE. In this regard, it cannot be discarded a delayed elimination of VLDL/LDL from plasma by microvascular endothelium [43]; although the characteristics of lipoproteins and vessels required for this process to occur are still unknown. Hence, specific studies are needed to define whether fibrates alter endothelium permeability or impede lipoproteins leakage through vessels.

Recent evidence demonstrated a slight increase in CETP activity with long-term pitavastatin treatment [47]. In addition, fenofibrate treatment upregulates the expression of CETP [48]. Then, the huge increase of the bidirectional CE flow rates between lipoproteins with the atorvastatin, fenofibrate, and the combination treatments presupposed an increased CETP activity. However, the activity of this protein was not modified by any of the treatments. The lack of increase in the CETP activity in our study may be related to the structural differences between pitavastatin and atorvastatin. In the case of fenofibrate, the increase in the CETP activity was demonstrated in CETP-transgenic mice (a species non-expressing CETP); it is possible that the CETP promoter of transgenic mice differs from that of rabbits, thus explaining the lack of the increase of CETP activity. Independently of the mechanism, we found that the CETP activity was not responsible for the over-dynamic transfer of CE between VLDL/LDL and HDL.

It is important to emphasize that more than 90% of the cholesterol exchanged between HDL and LDL is CETP-independent [49,50]. Therefore, the potential underlying mechanism of the exacerbated exchange of CE between lipoproteins may be related with the structure of HDL (i.e., size, lipid composition, and/or fluidity of HDL) [24,51,52]. In this regard, the size of HDL (determined by densitometry and the maximal point of absorbance) was increased in the rabbits treated with the combination and to a lesser extent in the rabbits treated with atorvastatin or fenofibrate. These observations agree with the higher relative proportion of the large HDL2b subclass in the rabbits treated with the combination of atorvastatin and fenofibrate. Since the interfacial flux of cholesterol between HDL2 and LDL is faster than that of HDL3 to LDL [49], it could be postulated that the increased size of HDL observed in the three groups of treated rabbits, enhances the transfer of CE between lipoproteins. This postulate is congruent with the enhanced bidirectional flux of CE between VLDL/LDL and HDL reported herein.

Another intrinsic characteristic of HDL that may affect the transfer of cholesterol between lipoproteins is the fluidity of the particle [24,52,53]. Considering that the larger the size of the lipoprotein, the higher the fluidity of their surface [53]; the increased size of HDL in rabbits treated with atorvastatin, fenofibrate, and the combination, supports the idea of more fluid HDL particles. In addition, fluidity of HDL particles is also determined by the degree of saturation of the acyl chains of their lipid components; in this context, our results clearly demonstrated that the proportion of saturated fatty acids (palmitic 16:0 and stearic 18:0) in HDL decreased, whereas unsaturated fatty acids (oleic 18:1 and linoleic 18:2) increased with any of the three treatments used in this study. Consequently, a higher proportion of more fluid acyl chains in the lipid moiety of HDL agrees with the increased transfer of cholesterol between VLDL/LDL and HDL [49,50].

Atorvastatin and fenofibrate have opposite effects on stearoyl-CoA desaturase-1 (SCAD-1), a key enzyme in the conversion of palmitic and stearic acids to palmitoleic (16:1) and oleic acids, respectively. Atorvastatin downregulates the expression of SCAD-1 [54], whereas fenofibrate increases their expression and activity [55,56]. On the other hand, the linoleic acid is not synthesized by animal cells, however, it is a component of several lipids within HDL [57]. Therefore, the increased relative amount of oleic and linoleic acids in detriment of palmitic and stearic acids in HDL of treated rabbits may not be related to an improved endogenous synthesis. Instead, this observation evokes an improved absorption of unsaturated fatty acids or a limited degradation of oleic and linoleic acids. Further studies in this field are needed to elucidate the specific mechanism that leads to an increased proportion of unsaturated fatty acids within HDL during the treatment with atorvastatin and fenofibrate.

Finally, we recognize as a limitation of our study the lack of results concerning the excretion of cholesterol in feces, which is considered the final step of RCT. We focused only on intravascular HDL-CE kinetics since it is known that atorvastatin stimulates cholesterol elimination in feces, not via HDL but mainly via hepatic LDL clearance [58]. Similarly, fenofibrate decreases fecal elimination of cholesterol, probably by downregulating cholesterol 7-alpha-hydroxylase and sterol 27-hydroxylase expression [59].

## 4. Materials and Methods

### 4.1. Animals

Male New Zealand white rabbits weighing 2.5 to 3 kg were randomized into four different groups and were treated with an oral daily dose of 0.33 mg/kg of atorvastatin, 2.6 mg/kg of fenofibrate, or the combination of both drugs for 8 weeks. The rabbits that only received the vehicle were included as a control group. The rabbits had free access to a normal chow diet and water ad libitum.

All procedures were performed in accordance with the “Guide for the Care and Use of Laboratory Animals” [60] and approved by the Scientific and ethics Committee from the Instituto Nacional de Cardiología “Ignacio Chávez”, with the project name “Effect of the atorvastatin and fenofibrate combination on the metabolism and antiatherogenic properties of HDL”, in 27 April 2012, with the number 12-764-Atorvastatina.

### 4.2. Blood Samples

Ten-hour fasting blood samples were drawn from the central artery of the ear in tubes with anticoagulant (15 UI/mL, sodium heparin). Blood samples were centrifuged for 15 min at 1300× *g*. The plasma was separated into 1 mL aliquots and was frozen at −70 °C until use.

### 4.3. Biochemical Analyses

The total cholesterol, triglycerides, and glucose plasma concentrations were determined by enzymatic colorimetric methods (Randox Laboratories, Antrim, UK). The phosphotungstic acid-Mg^2+^ method (Randox Laboratories, Antrim, UK) was used to precipitate the apo B-containing lipoproteins (i.e., VLDL/LDL, very low-density lipoproteins/low-density lipoproteins) in the plasma. In the supernatant fraction, HDL-C, high-density lipoproteins-triglycerides (HDL-Tg), and high-density lipoproteins-phospholipids (HDL-Ph) plasma concentrations were determined by enzymatic colorimetric methods (Randox Laboratories, Antrim, UK and Wako Chemicals, Richmond, VA, USA). Apo A-I plasma concentration was determined by a commercial ELISA kit (Mybiosource, San Diego, CA, USA). All the determinations were performed following the instructions of the manufacturers.

### 4.4. Isolation of HDL

HDL were isolated as previously reported [61]. Briefly, 1 mL of plasma was adjusted to a density (δ) of 1.063 g/mL with solid KBr in polycarbonate tubes and centrifuged for 2.5 h at 10 °C and 543,000× *g*. The supernatant fraction was separated and the total volume of plasma in the remaining fraction was adjusted to δ 1.21 g/mL with KBr and was centrifuged for 3 h at 10 °C and 543,000× *g*. HDL were recovered from the supernatant fraction and were centrifuged for 3 h at 10 °C and 543,000× *g* with 1.25 g/mL KBr density solution. Finally, HDL were dialyzed against 0.09 mol/L tris/0.08 M boric acid/3 mM EDTA buffer, pH 8.4.

### 4.5. Determination of HDL Lipid Composition

HDL size distribution was estimated as previously reported [28]. Briefly, 25 μg of HDL protein per well were separated in a non-denaturing 3%–30% gradient polyacrylamide gel electrophoresis. The electrophoresis was performed for 22 h at 180 V. The gels were stained for cholesterol, triglycerides, and phospholipids using in-house prepared enzymatic mixtures [62,63]. The gels were incubated for 30 min at 37 °C in darkness, washed with water, and were scanned to obtain an image (lipid image) in a GS-670 densitometer (Bio-Rad laboratories, Hercules, CA, USA). Thereafter, gels were distained with 25% methanol/10% acetic acid/65% water solution and were re-stained for protein with 0.1% Coomassie blue R-250 (Bio-Rad laboratories, Hercules, CA, USA) solution. Again, the gels were scanned to obtain a new image (protein image). The size of HDL (Stoke’s diameter) subclasses was determined by densitometric analysis considering the following size intervals: HDL 3c, 7.94–8.45 nm; HDL 3b, 8.45–8.98 nm; HDL 3a, 8.98–9.94 nm; HDL 2a, 9.94–10.58 nm; HDL 2b, 10.58–13 nm. The densitometric analysis was performed using as reference a high-molecular weight calibration kit of globular proteins (Amersham Pharmacia Biotech, Buckinghamshire, UK) and the Molecular Analyst software (Bio-Rad laboratories, Hercules, CA, USA). The size of HDL was determined by seeking the maximal point of absorbance in the HDL graphs. The relative proportion of protein or lipid of the HDL subclass was represented as the percentage of the area under the curve of each size interval with respect to the total area under the curve determined by the densitometric analysis. HDL-C, HDL-Tg, and HDL-Ph plasma concentrations of each HDL subclass were calculated as previously reported [62,63].

### 4.6. Determination of Fatty Acids Composition of HDL

Fatty acids composition was determined by gas chromatography–mass spectrometry (GC-MS) as previously reported [64] with some modifications. Briefly, total lipids were extracted from isolated HDL and the fatty acids were esterified using anhydrous benzene/methanol solution (1:1, *v/v*) and concentrated sulfuric acid. The esterified fatty acids were extracted with hexane and it was evaporated with a flow of N_2_. Five µl of ethyl oleate (1:50 in hexane as internal control, Sigma-Aldrich, St Louis, MO, USA) and 15 µl of hexane were added to the esterified fatty acids samples and 0.8 µl were injected into the chromatograph. Fatty acids were separated and analyzed in a Hewlett Packard 5972 GC-MS system using the following conditions: capillary column coated with polyethylene glycol (30 m length, 0.32 mm diameter, and 0.5 µm film thickness, HP-INNOWAX 19091N-213, Hewlett Packard, Palo Alto, CA, USA), helium as a mobile phase (pressure 244 kPa), split flow at 15 mL/min, temperature of injector at 240 °C, detector at 260 °C, oven initial temperature at 170 °C, and final temperature 260 °C, and a total run time of 21 min. The conditions were standardized with a commercially available mixture of fatty acids methyl esters SUPELCO FAME MIX GLC-10, GLC-20 and GLC-50 (Sigma-Aldrich, St Louis, MO, USA).

### 4.7. Preparation of Labeled HDL with [^3^H]-Cholesterol

Ten milliliters of plasma from donor rabbits were adjusted to δ 1.063 g/mL and were centrifuged as described above. The supernatant fraction was discarded and the total volume of plasma in the remaining fraction was dialyzed against 150 mM NaCl/8.6 mM Na_2_HPO_4_/1.4 mM NaH_2_PO_4_ buffer (PBS), pH 7.4. The volume of plasma recovered was measured and was placed in a glass tube. Afterwards, the tube with plasma was tempered at 37 °C. The plasma was labeled with 1 μCi/mL of tritiated cholesterol (specific activity 40–60 Ci/mmol, American Radiolabeled Chemicals Inc, St. Louis, MO, USA) and was incubated for 18 h at 37 °C with constant stirring to allow the esterification of tritiated cholesterol within the HDL by lecithin-cholesterol acyltransferase. After the incubation period, the HDL-[^3^H]-CE were isolated by ultracentrifugation. The HDL-[^3^H]-CE were passed through a 1 × 10 cm desalting column packed with Sephadex G-25 (50–150 μ, Pharmacia Fine Chemicals, Piscataway, NJ, USA) to remove the remaining free cholesterol. The fractions with the highest concentrations of cholesterol and protein were recovered and were pooled. Finally, the HDL-[^3^H]-CE were filtered through a 0.22 μm Millex-GV filters (Millipore, Burlington, MA, USA) and were refrigerated until use.

### 4.8. In Vivo Kinetic Studies of HDL-[^3^H]-Cholesteryl Esters

A bolus equivalent to 1 × 10^6^ counts per minute of HDL-[^3^H]-CE were injected into the left marginal ear vein. Blood samples of 1 mL were drawn from the opposite marginal ear vein at 5, 15, 30, 45, 60, 90, 120, 150, 180, 240, and 300 min after the injection of the bolus. The blood samples were collected into tubes containing 15 UI/mL sodium heparin and were kept on ice until use. The blood samples were centrifuged for 15 min at 1300 g and 4 °C. Then, the VLDL/LDL (δ 1.063 g/mL) and the HDL (δ 1.21 g/mL) fractions were isolated by ultracentrifugation as mentioned above. Radioactivity in both fractions, VLDL/LDL and HDL, was measured in a liquid scintillation analyzer TRI-CARB 2200CA (Packard Instruments, Downers Grove, IL, USA).

### 4.9. Compartmental Analysis

**Figure 3:**
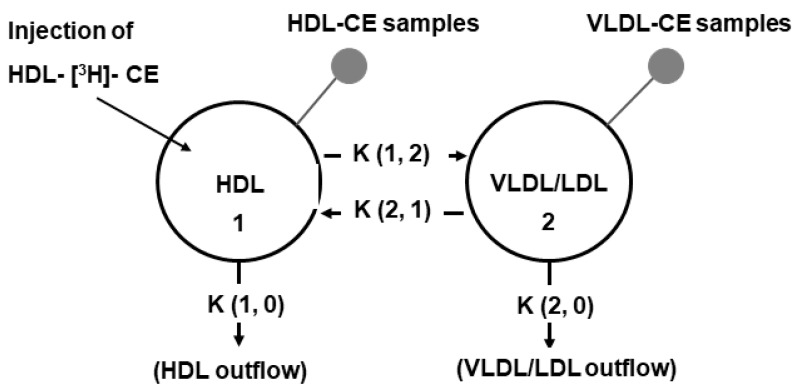
Compartmental model for the transfer of CE between lipoproteins. Circles represent the two lipoproteins compartments and the arrows represent the direction of the transfer or the elimination pathway of cholesteryl esters. Full circles represent the compartment where the samples were obtained.

The radioactivity data were mathematically fitted to a two-compartment model. For the model (Figure 3), we considered two main plasma compartments; compartment 1 corresponding to the HDL fraction, whereas the VLDL/LDL fraction constituted compartment number 2. Additionally, four transfer coefficients were considered in the model (Figure 3); the transfer of CE from the HDL to VLDL/LDL (pro-atherogenic transfer) was represented by the transfer coefficient K (1, 2), the transfer of CE from the VLDL/LDL to HDL (anti-atherogenic transfer) was represented by the transfer coefficient K (2, 1) and the outflow of CE via HDL and VLDL/LDL were represented by the transfer coefficients K (1, 0) and K (2, 0), respectively. Modeling of the data was done with the SAAM II software (SAAM Institute, Seattle, WA, USA). The flow rates were calculated by multiplying the plasma concentration of CE by their corresponding transfer coefficient. The compartmental analysis was based upon a previous report [29].

### 4.10. Cholesteryl Ester Transfer Protein Activity Assay

The activity of the cholesteryl ester transfer protein (CETP) was determined from plasma as previously reported [65]. Briefly, the VLDL/LDL fraction (δ 1.063 g/mL) was isolated by ultracentrifugation from a pool of plasma of five healthy human donors. The pool was then labeled with 1 µCi/mL of tritiated cholesterol and the [^3^H]-HDL3 fraction (δ 1.12 g/mL) was isolated by ultracentrifugation. For the CETP assay, 3 μL of the [^3^H]-HDL3 (at 40 mg/mL of protein), 100 μL of VLDL/LDL (at 2.5 mg/mL of protein), and 10 μL of rabbit plasma were mixed in 500 μL of 10mM Tris/150 mM NaCl/2 mM EDTA/0.01% NaN_3_ solution, pH 7.4. All mixture samples were incubated for 16 h at 37 °C. Thereafter, total radioactivity in mixture samples was measured in a liquid scintillation analyzer. The reaction was stopped with 50 μL of 10 g/L dextran sulfate/0.5 M MgSO_4_ solution to precipitate VLDL/LDL and radioactivity in the supernatant was counted. The CETP activity was calculated as the percentage of radioactivity transferred from HDL3 to VLDL/LDL fraction by 10 μL of rabbit plasma during the 16 h. Control plasma and reactive blank were included in each assay and all samples were determined twice.

### 4.11. Statistical Analysis

Normal distribution of data was determined by the Kolmogorov–Smirnov test. Data with normal distribution were expressed as mean ± standard error and comparisons between groups were performed using the one-way ANOVA test. Data without normal distribution were expressed as median and interquartile range and comparisons between groups were made with the Kruskal–Wallis test. The IBM Statistical Package for the Social Sciences (SPSS) version 21 software for Windows (International Business Machines Corp., Armonk, NY, USA) was used to perform the statistical analyses. Comparisons were considered statistically different at *p* value < 0.05.

## 5. Conclusions

We demonstrated significant modifications of the HDL structure induced by atorvastatin, fenofibrate, and the combination of both drugs, concomitantly with an improved transfer of CE between lipoproteins. Our results suggest that the structure of HDL is a main determinant of CE kinetics in vivo, and also suggest a potential synergic effect between the atorvastatin and fenofibrate. These results contribute to a better understanding of the relationship between the structure and function of HDL during the use of anti-dyslipidemic drugs.

## Figures and Tables

**Table 1:** Biochemical analyses.

Biochemical Parameters	Control *n* = 6	Atorvastatin *n* = 6	Fenofibrate *n* = 6	Combination *n* = 6
Cholesterol (mmol/L)	1.41 ± 0.06	1.19 ± 0.11	1.26 ± 0.17	1.30 ± 0.07
Triglycerides (mmol/L)	0.75 ± 0.05	0.93 ± 0.23	0.66 ± 0.08	0.75 ± 0.11
Glucose (mmol/L)	6.25 ± 0.32	6.28 ± 0.14	6.39 ± 0.28	6.10 ± 0.10
HDL-C (mmol/L)	0.79 ± 0.03	0.79 ± 0.05	0.89 ± 0.10	1.14 ± 0.12 *^,^**^,^***
HDL-Tg (mmol/L)	0.42 ± 0.06	0.44 ± 0.06	0.37 ± 0.02	0.46 ± 0.10
HDL-Ph (mmol/L)	2.47 ± 0.22	2.39 ± 0.10	2.32 ± 0.23	3.07 ± 0.27 **^,^***
Apo A-I (mg/L)	398.6 ± 32.6	408.1 ± 20.8	617.6 ± 10.8*	560.2 ± 77.0 *

HDL-C, high-density lipoprotein-cholesterol; HDL-Tg, high-density lipoprotein-triglycerides; HDL-Ph, high-density lipoprotein-phospholipids; Apo A-I, apolipoprotein A-I. Data are mean ± standard error. ANOVA test, *p* < 0.05 vs. * control, ** atorvastatin, and *** fenofibrate.

**Table 2:** Cholesterol, triglycerides, and phospholipids plasma concentrations of HDL subclasses.

HDL Subclasses	Control *n* = 6	Atorvastatin *n* = 6	Fenofibrate *n* = 6	Combination *n* = 6
C (µmol/L)				
HDL2b	411.1 ± 36.9	416.2 ± 38.3	438.2 ± 61.3	586.1 ± 90.8 *
HDL2a	141.4 ± 3.5	144.3 ± 8.9	154.8 ± 19.4	199.2 ± 29.3 *^,^**
HDL3a	181.5 ± 12.4	178.0 ± 19.8	201.3 ± 28.4	245.9 ± 28.3**
HDL3b	44.9 ± 8.6	38.8 ± 7.4	67.4 ± 8.2	64.4 ± 13.6
HDL3c	12.4 ± 3.3	11.3 ± 2.3	29.9 ± 3.8	41.3 ± 24.7
Tg (µmol/L)				
HDL2b	206.3 ± 32.4	212.9 ± 38.9	172.1 ± 15.9	208.5 ± 35.9
HDL2a	71.9 ± 10.8	74.8 ± 9.7	64.1 ± 5.8	75.6 ± 15.8
HDL3a	101.4 ± 16.1	106.1 ± 13.9	93.2 ± 5.3	116.2 ± 28.1
HDL3b	30.2 ± 6.6	33.6 ± 11.9	29.7 ± 6.4	43.0 ± 14.1
HDL3c	8.1 ± 3.6	14.3 ± 6.8	12.2 ± 4.2	21.7 ± 12.1
Ph (µmol/L)				
HDL2b	1347.8 ± 145.4	1308.6 ± 78.8	1273 ± 147.2	1826.2 ± 164.8 *^,^**^,^***
HDL2a	411.2 ± 29.4	414.9 ± 15.9	403.7 ± 31.8	473.5 ± 33.9 *^,^**^,^***
HDL3a	527.4 ± 44.5	521.0 ± 35.2	513.7 ± 37.4	572.7 ± 60.5
HDL3b	147.6 ± 19.5	108.6 ± 16.8	103.5 ± 17.1	148.2 ± 34.2
HDL3c	38.8 ± 6.0	35.6 ± 6.5	25.2 ± 7.2	50.9 ± 14.9

C, cholesterol; Tg, triglycerides; Ph, phospholipids. Data are mean ± standard error. ANOVA test, *p* < 0.05 vs. * control, ** atorvastatin, and *** fenofibrate.

**Table 3:** Transfer rates of cholesteryl esters between lipoproteins.

Constants	Transfer of CE	Control *n* = 6	Atorvastatin *n* = 6	Fenofibrate *n* = 6	Combination *n* = 6
K (1, 2)	HDL to VLDL/LDL	10.60 (8.09–12.29)	71.30 * (43.45–84.11)	32.74 (9.23–64.87)	38.92 * (21.65–51.77)
K (2,1)	VLDL/LDL to HDL	11.46 (5.19–22.16)	80.63* (52.42–101.25)	23.37 (12.62–68.91)	82.81 * (10.55–190.81)
K (1, 0)	HDL outflow	3.28 (3.06–5.14)	4.69 (3.22–6.70)	2.34 (1.66–5.56)	5.69 * (5.20–6.31)
K (2, 0)	VLDL/LDL outflow	8.08 (5.07–9.55)	7.26 (6.67–9.19)	3.94 *^,^ ** (2.22–4.98)	5.83 (3.89–18.91)

Data are median and interquartile range. Transfers of cholesteryl esters (CE) mass are in µmol/L per min. Kruskal–Wallis test, *p* < 0.05 vs. * control and ** atorvastatin.

## Abbreviations

HDL-C	High-density lipoproteins-cholesterol
HDL	High-density lipoproteins
Apo A-I	Apolipoprotein A-I
CE	Cholesteryl esters
RCT	Reverse cholesterol transport
HDL-CE	High-density lipoproteins-cholesteryl esters
VLDL/LDL	Very low-density lipoproteins/low-density lipoproteins
HDL-Tg	High-density lipoproteins-triglycerides
HDL-Ph	High-density lipoproteins-phospholipids
GC-MS	Gas chromatography–mass spectrometry
CETP	Cholesteryl ester transfer protein
